# When breast cancer patients participate in ritual interactive activities: the mechanism of perceived emotional synchrony on health information avoidance

**DOI:** 10.3389/fpsyg.2025.1566773

**Published:** 2025-07-31

**Authors:** Jie Chen, Yang Yang, Fangjuan Du, Jie Li

**Affiliations:** ^1^School of Geography and Environmental Sciences (School of Karst Science), Guizhou Normal University, Guiyang, China; ^2^School of Tourism and Air Service, Guizhou Minzu University, Guiyang, China; ^3^School of International Tourism and Culture, Guizhou Normal University, Guiyang, China; ^4^School of Geographical Sciences and Remote Sensing, Guangzhou University, Guangzhou, China

**Keywords:** perceived emotional synchrony, positive emotions, coping self-efficacy, health information avoidance, breast cancer, a moderated chain mediation model

## Abstract

**Introduction:**

Health information avoidance (HIA) creates serious health risks, particularly for patients with serious health problems such as breast cancer. Although existing research has explained how emotional responses affect HIA from several perspectives, little attention has been paid to how perceived emotional synchrony (PES), as an antecedent, influences HIA behavior, especially in the context of breast cancer patients participating in ritualistic interactive activities. In this study, we constructed a moderated chain mediation model drawn on the Interactive Ritual Chains (IRCs) theory, combined with social cognitive theory to test the relationship between PES and HIA behaviors in cancer patients. At the same time, the important individual characteristic of cancer staging has been overlooked in studies of boundary mechanisms in HIA. We further explored the moderating role of cancer staging.

**Methods:**

We assembled a sample of 302 female patients with breast cancer who participated in ritual interaction activities in five Grade A tertiary hospitals in China. In this study, regression analysis was conducted using SPSS 23.0 and MPlus 8.3 to explore the relationship between PES, positive emotions, coping self-efficacy, and HIA variables to test the hypotheses.

**Results:**

Empirical analyses revealed that PES was negatively correlated with HIA in the context of breast cancer patients participating in ritual interaction activities. Additionally, positive emotions and coping self-efficacy acted as mediators between PES and HIA. Furthermore, positive emotions and coping self-efficacy played a chain-mediation role in the relationship between PES and HIA during ritual interaction activities. Disease stage significantly moderated the strength of these chain-mediated effects, with the chain-mediated influence of positive emotions and coping self-efficacy between PES and HIA being significantly stronger in patients with advanced breast cancer.

**Discussion:**

The study constructed a quantitative conceptual model of how PES influences HIA in cancer patients. Cancer staging was shown to have a moderating effect on this mechanism, which enriches theoretical explanations of HIA behavior. In practice, promoting PES through structured ritual interactions can strengthen emotional connections among breast cancer patients. Developing stage-specific support strategies may facilitate more personalized interventions. Future research should examine the multilevel mechanisms of ritual interaction and the situational role of HIA.

## 1 Introduction

Breast cancer is a major global health concern (Smit et al., 2019; Wilkinson and Gathani, 2022). According to the recent global cancer burden figures, it is estimated that there were 2.3 million incident breast cancer cases in 2022, making it the most prevalent malignant tumor in the world; it is also a leading cause of cancer-related mortality among women (World Health Organization, 2024). Timely and accurate access to health information is critical for effective diagnosis, treatment, treatment decision-making, and recovery processes in cancer care (Anker et al., 2011; Lu et al., 2024). Nonetheless, a substantial proportion of breast cancer patients make deliberate efforts to avoid cancer-related information that may induce emotional distress or perceived threat. For instance, Melnyk and Shepperd (2012) reported that nearly 25% of women were reluctant to confirm a cancer diagnosis. Furthermore, recent studies indicate that numerous patients actively avoid cancer-related topics (Calderon et al., 2021; Nelissen et al., 2015; Yu et al., 2015). Such health information avoidance (HIA) behaviors have been shown to lead to delayed diagnosis, decreased adherence to treatment regimens, and ultimately poorer health outcomes (Golman et al., 2017; Howell et al., 2020; Link, 2022; Lu et al., 2024; Melnyk and Shepperd, 2012). Understanding the psychological and social mechanisms behind HIA is critical for improving patient engagement and health outcomes in cancer care.

Past research has focused on multiple factors that influence HIA: first, individual differences are manifested in health perceptions (Emanuel et al., 2015; Lipsey and Shepperd, 2019), perceived control (Brown et al., 2017), and emotional motivation (Emanuel et al., 2015; Hertwig and Engel, 2016). Second are social factors, such as social relationships (Howell and Shepperd, 2017; Link and Baumann, 2022; Lipsey and Shepperd, 2019; Price, 2018) and socioeconomic status (Pampel et al., 2010). Finally, information factors relate more to information content (Ganguly and Tasoff, 2017) and information overload (Franconeri et al., 2013; Siebenhaar et al., 2020). A supportive environment and peer interaction provide a unique sense of community and belonging to patients with cancer and meet their perceived emotional needs [e.g., patients' emotional synchrony perception (PES)], while helping patients access cancer treatment information and linking to resources (Ussher et al., 2006). However, research on PES as a factor in the emotional motivation affecting HIA is less frequently conducted, especially in the context of breast cancer. The term PES refers to the idea that people experience similar emotions when participating in rituals, group activities, or synchronized behaviors (Durkheim, 1915; Wlodarczyk et al., 2020; Xiang et al., 2022b). This feeling reflects emotional resonance and a perceived sense of connection and unity among individuals (Collins, 2004; Gabriel et al., 2017; Wood, 2020). PES also produces positive affective outcomes such as increased trust, belonging, and group cohesion (Cheong et al., 2023; Páez et al., 2015; Pizarro et al., 2020). Zlobina and Dávila (2022) emphasize that collective PES experiences and cultural practices have a positive impact on public health behaviors during times of crisis. For emotionally vulnerable populations such as cancer patients, PES may serve as an important emotional buffer that enhances psychological resilience and motivates more open engagement with distressing but necessary health information. Nevertheless, its potential as a protective emotional factor against health information avoidance in oncology contexts has yet to be adequately addressed.

Scholars have explained how emotional responses affect HIA from the following perspectives. Soroya et al. (2021) applied the stimulus–organism–response (SOR) model and introduced an affective layer to explain how information exposure can lead to avoidance behavior during the COVID-19 pandemic. Zhang and Gao (2021) used the cognition–affect–conation model to study how anxiety affects users' information avoidance and withdrawal on health platforms. In another approach Deline and Kahlor (2019) proposed the Program Risk Information Avoidance (PRIA) model that incorporates cognitive, affective, and sociocultural dimensions. Other studies have drawn on drive theory and the elaboration likelihood model to explore similar avoidance behaviors. However, the above studies have paid little attention to how PES, as an antecedent, influences HIA behavior through emotions and cognition, especially in contexts where patients with breast cancer engage in ritual interaction activities. To address these gaps, the Interactive Ritual Chains (IRCs) theory, proposed by Collins (2004), was introduced to explain the emotional energy of patients with cancer when they engage in ritual interaction activities (Ritchie and Hudson, 2009). According to IRCs, scholars have suggested that participants' shared concerns and emotions trigger reciprocal neurological arousal (Collins, 2004) and have emphasized that higher PES in collective gatherings leads to stronger emotional responses (Páez et al., 2015). The emotional response and regulatory abilities of individuals have been shown to play a crucial role in explaining why people engage in HIA (Yang and Kahlor, 2013; Chasiotis et al., 2021; Yang et al., 2023). Combined with social cognitive theory, perceived emotional and physiological states affect individual self-efficacy (Bandura, 1998), and whether an individual develops HIA behavior is a consequence of changes in self-efficacy (Hua and Howell, 2022). Accordingly, we argue that positive emotions and cognition (e.g., coping self-efficacy) may be associated with HIA behavior, especially in states where participants' emotional energy is mutually aroused. The IRCs theory proposes that social interactions generate emotional energy and cognitive value, which in turn foster shared emotional experiences (Collins, 2004; Yu and Na, 2022). When considered alongside social cognitive theory, the perception of emotions has been shown to influence individual self-efficacy (Bandura, 1998). Positive emotions have been demonstrated to enhance coping abilities and adaptation (Guo and Wang, 2007), while higher levels of self-efficacy have been found to increase patients' willingness to engage with health information (Lee et al., 2008). Fredrickson and Branigan (2005) emphasized that positive emotions form the foundation for building cognitive resources and behavioral capacities. Consequently, positive emotion and self-efficacy may act as mediating factors, linking PES to reduced HIA. This provides a theoretical foundation for exploring this mechanism. However, a chained explanatory mechanism explaining the relationship between PES and HIA in terms of the two constructs of positive affect and coping self-efficacy is lacking.

Scholars have also explained the boundary mechanisms of HIA in terms of situational and subjective factors. Contextual factors include cross-cultural situations, family, and informational environments. Subjective factors are mainly related to demographic characteristics and individual resources such as self-affirmation (Howell and Shepperd, 2017) and time pressure (Guo et al., 2020). However, disease progression stage (cancer staging) is an important individual characteristic that affects the mood and cognition of patients with cancer (Hou et al., 2018; Segrin et al., 2011); this characteristic has, to date, been missing in HIA studies. Therefore, the current study further considered the moderating role of cancer staging by comprehensively analyzing the relationship between the PES and HIA in patients with cancer.

Considering the discussion above, this study mainly aimed to (1) show the promoting influence of PES in patients with breast cancer's interaction on HIA behavior, (2) examine the chain-mediating role of positive emotion and coping self-efficacy, and (3) test the moderating effect of cancer staging. To reach these objectives, this study draws on IRCs theory, combined with social cognitive theory, and uses quantitative data collected from five Grade A tertiary hospitals in China to conduct an empirical study. Current research indicates that this is the first effort to verify the causal implications of PES in the ritual interactions of patients with cancer with HIA, quantitatively. We anticipate that the findings of this study will theoretically enrich research on the interactions and health behaviors of patients with cancer and provide reference points to address patients' HIA in practice.

### 1.1 The IRCs theory

Originally developed by Collins (1993, 2004), the theory of IRCs conceptualizes human interaction as a sequence of emotionally charged encounters, in which shared emotions and mutual focus are central. According to Collins (2004), a core outcome of successful rituals is emotional energy—a sustained feeling of enthusiasm, confidence, and initiative. Most interactive rituals produce the following outcomes: (1) group solidarity, (2) personal emotional energy, (3) symbols representing the group, and (4) morality. These rituals can take the form of either natural rituals, which are informal and embedded in everyday interactions, or formal rituals, which involve structured, symbolic activities (Collins, 2004).

Although IRC theory does not explain the direction of causality among constructs, it has proven to be of significant value in health research. Scholars have used it to explore how emotional energy generated through social interaction fosters behavioral transformation. For instance, Henckes and Nurok (2015) demonstrate that ritualized emotional exchanges among healthcare professionals foster a sense of belonging and promote adaptive behavior. Similarly, ritual interactions in the daily routine influence clinicians' behavioral decisions (Szymczak, 2016). Recent research has extended this framework to patient contexts. Clarke and Waring (2018) drew on the IRC theory to determine how interactions can help individuals transform negative emotions into positive ones. Their findings indicated that ritual interactions lead to positive results for patients, such as solidarity, belonging, confidence, and positive change. In epidemic settings, Xiang et al. (2022a) explored how ritualized “host–guest” exchanges contributed to emotional regulation and value construction. While prior studies were mainly qualitative, they provide critical conceptual insights into how ritual interactions generate emotional energy and shape behavioral outcomes. These insights provide a solid basis for identifying and operationalizing key constructs such as PES. Based on this foundation, Zlobina and Dávila (2022) conducted empirical research and found that emotional synchrony during collective ritual participation significantly and positively predicts preventive behaviors through cognitive-affective processes. This finding offers direct support for the quantitative measurement of key IRC constructs, such as PES, and their prediction of behavior. This approach enables us to advance beyond descriptive accounts and empirically test the pathways through which ritual participation influences health-related behaviors.

However, the IRCs theory has not been widely used to show how emotional energy generated through ritual interactions may influence health-related behaviors, such as HIA, particularly in the context of breast cancer patients. In this study, we mainly use IRCs theory as a conceptual lens to understand the emotional dynamics that shape PES in patient ritual interactions. Additionally, we integrate this framework with social cognitive theory (Bandura, 1997, 1998) to support the proposed links between PES, coping self-efficacy, and behavioral responses in the context of breast cancer care.

### 1.2 Patients' PES and HIA behavior

The term PES means how people feel emotionally connected and share feelings when they take part in ritual interactions (Páez et al., 2015; Wlodarczyk et al., 2020). Instead of being a specific emotion, it is better seen as a mental process. The phenomenon represents a strongly shared emotional experience (Durkheim, 1915; Xiang et al., 2022b). PES may trigger collective affective states during group gatherings, shared attention, and when behavioral synchrony occurs (Wlodarczyk et al., 2020). PES reflects the dynamic interactions between event participants and between hosts and guests (Sterchele, 2020; Xiang et al., 2022b; Xie and Li, 2023). Emotional synchrony is a psychological element that provides a sense of connection to others and a sense of belonging to a group (Collins, 2004; Gabriel et al., 2017). This experience typically leads to positive outcomes such as trust, safety, and emotional support (Páez et al., 2015).

On the other hand, avoiding information is described as “denial, blunting, or repression” (Lambert and Loiselle, 2007); it refers to a person's deliberate choice to ignore or distance themselves from the information (Zhao and Liu, 2021). Information avoidance is strongly correlated with negative emotional states such as anxiety, fear, which often arise when individuals feel emotionally overwhelmed or socially isolated (Case et al., 2005). Research on the psychology of emotions has demonstrated that the greatest motivator of behavior is emotion (Lazarus, 1991; Zeelenberg et al., 2008), which motivates health information seeking (Myrick et al., 2016), and reduces HIA. In this context, PES may help patients regulate their negative emotions by fostering emotional safety, reducing feelings of threat, and creating a socially supportive environment. Shared emotional experiences can help individuals feel less isolated and more confident when facing difficult health realities. For example, in group settings such as patient support groups or rituals, emotional synchronization can buffer distress and increase patients' willingness to confront and process health-related information. Deline and Kahlor (2019) pointed out that affective factors can influence information avoidance in risky situations. They also stressed that reducing negative emotions can increase information engagement. Thus, PES can be considered a protective affective factor that may reduce the chance of HIA by promoting emotional openness, psychological resilience, and group-based coping. Based on this reasoning, the following hypothesis is proposed:

*H1. PES of patient interactions has a significant negative effect on HIA behavior*.

### 1.3 Mediating effect of positive emotion

Positive emotions are unique immediate responses to personally meaningful and temporary pleasure (Fredrickson, 2001). They are an important driver of individuals' behavioral intentions (Fredrickson and Branigan, 2005) and a core measure of personal health behaviors (Folkman and Moskowitz, 2000; Mayne and Bonanno, 2001). IRC theory suggests that PES in group gatherings is associated with emotional responses (Collins, 2004; Durkheim, 1915). Páez et al. (2015) verified that higher PES resulted in more positive emotions.

In this study, we argue that the emotional energy produced during cancer patients' ritual interactions can activate specific emotional responses, such as emotional arousal and empathy. These emotional responses contribute to the emergence of positive emotions (e.g., hope, trust, relief) that are shaped by shared affective experience (Gabriel et al., 2017; Xiang et al., 2022b). Therefore, the related hypotheses are as follows:

*H2a. PES of patient interaction has a significant positive effect on patients' positive emotions*.

In a positive emotional state, individuals tend to approach and explore new things, and maintain an active connection with the environment. Positive emotions are linked to specific action tendencies, such as generating exploration and grasping new information and experiences (Fredrickson and Branigan, 2005). Yang and Kahlor (2013) indicated that affective factors are correlated with information avoidance behaviors under severe health concerns. A clinical trial has verified that positive emotions have the potential to stimulate more positive information seeking (Yang et al., 2011). Hence, we infer that:

*H2b. Positive emotions of patients have a significant negative effect on HIA behavior*.

Health sociology scholars have discussed the antecedents and outcome variables of patients' positive emotions. The antecedent variables affecting positive emotions include physical environment (e.g., healthcare setting) and social environment (e.g., hospitableness and social interactions) (Altinay et al., 2023; Batra and Taneja, 2022). The outcome variables included satisfaction, adherence, subjective well-being, and behavioral intentions (Altinay et al., 2023; Legg et al., 2015). This study considers that patients' PES influences their HIA behavior, via positive emotions. According to IRCs theory, behavioral symbols in interaction have a strong cultural function, which can better link participants' emotional resonance and subsequent behaviors (Collins, 2004). As stated by Zhou et al. (2023), positive emotions are a connection mechanism for mental states that mediate social interactions and individual behaviors. Additionally, by combining H2a and H2b, we propose the following:

*H2. Positive emotions mediate the negative relationship between the PES of the patient and their HIA behavior*.

### 1.4 Mediating effect of coping self-efficacy

Coping self-efficacy is a psychological resource for helping patients adjust to breast cancer (Karademas et al., 2021, 2022), reflecting an individual's subjective judgment of their ability to cope effectively with challenges in life (Bandura, 1982; Chesney et al., 2006). In the context of cancer, coping self-efficacy is a context-specific form of self-efficacy that refers to a patient's perceived ability to cope with the challenges of cancer treatment (Chirico et al., 2017), such as uncertainty associated with the diagnosis of the disease, physical side effects of treatment, postoperative recovery, informational challenges of treatment (Heitzmann et al., 2011). Scholars distinguish three forms of coping self-efficacy: emotion-focused coping, problem-focused coping, and support from family and friends (Chesney et al., 2006), with emotion-focused coping receiving particular attention for its significant impact on emotional adjustment, especially in the context of serious illnesses such as breast cancer.

Coping self-efficacy refers to individuals' belief in their ability to effectively manage challenges, particularly in the context of illness, and it reflects human cognition (Bandura, 1997). According to social cognitive theory, such belief is shaped not only by personal experiences but also by emotional and social interactions (Bandura, 1998). Research has found that PES promotes emotional connectedness, which strengthens self-relevant evaluations such as self-efficacy (Páez et al., 2015). When confronted with stressful events, PES provides emotional support by sharing experiences with others, which may enhance individuals‘ confidence in coping with adversity, thereby increasing their coping self-efficacy (Hua and Howell, 2022; Lee et al., 2008). Therefore, the related hypothesis is as follows:

*H3a. PES of patient interaction has a significant positive effect on patient coping self-efficacy*.

Related research states that self-efficacy influences individuals to adopt behaviors and improve outcomes (Bandura, 1978, 1997). Coping self-efficacy is a personal resource that may play a crucial role in defensive decision-making, such as avoiding information (Marx-Fleck et al., 2021). Individuals have limited available resources, and when their perceived resources are diminished or insufficient (i.e., “threatened”), they respond to uncertainty and stress through avoidance motivation (Bublatzky et al., 2017). Lee et al. (2008) emphasized that patients' coping self-efficacy is a key factor in determining their use of health information. Insufficient coping self-efficacy may lead to information avoidance when individuals feel highly threatened (Hua and Howell, 2022; Lee et al., 2008; Witte, 1992). Therefore, the related hypothesis is as follows:

*H3b. High coping self-efficacy has a significant negative effect on HIA behavior*.

Self-efficacy is a personal resource that influences behavior (Abdullah and Wider, 2022; Bandura, 1978). Hence, coping self-efficacy has been identified as an effective mediator of health-promoting behaviors (Geng et al., 2018). For instance, Geng et al. (2018) suggested that good social support for patients with cancer positively influences information seeking indirectly, via coping self-efficacy. We therefore infer that coping self-efficacy is a mediating mechanism linking patients' PES and HIA behaviors. Therefore, by combining hypotheses H3a and H3b, we propose the following:

*H3. Coping self-efficacy mediates the negative relationship between patients' PES and HIA behaviors*.

### 1.5 The chain-mediating effect of positive emotions and coping self-efficacy

According to the IRC theory, social interactions activate emotional energy and cognitive value (Collins, 2004; Yu and Na, 2022). In combination with social cognitive theory, perceived emotions affect individual self-efficacy (Bandura, 1998). Guo and Wang (2007) noted that positive emotions can improve individuals' coping levels and promote social adaptation. The greater the patient's self-efficacy, the higher the chance of determining their use of health information services (Lee et al., 2008). In summary, by combining H2 and H3, the following hypothesis is proposed:

*H4. Patients' positive emotions and coping self-efficacy chains mediate the relationship between PES and HIA behavior*.

### 1.6 The moderating effect of cancer staging

Cancer staging is not only a medical indicator of disease progression but also has important psychological and behavioral implications. In oncology practice, breast cancer is staged using the internationally recognized TNM system. That categorizes cancer into stages 0, I, II, III, and IV based on tumor size (T), lymph node involvement (N), and presence of distant metastasis (M) (Amin et al., 2017). Generally, a higher stage means more advanced disease and poorer prognosis (Yao et al., 2015). However, in the Chinese clinical and cultural context, patients are often more familiar with simplified categories such as “early,” “middle,” and “advanced” stages, rather than specific TNM stages. To ensure both conceptual clarity and patient comprehension, based on recommendations from oncology professionals, field research (Manne et al., 2001; Segrin et al., 2011; Yao et al., 2015), and the 2022 edition of China's national Breast Cancer Diagnosis and Treatment Guidelines (National Health Commission of the People's Republic of China, 2022), this study adopts the following classification: the early stage includes stage 0, stage I, and part of stage II (IIA and IIB with T2N1M0); the middle stage includes stage IIB (T3N0M0) and stage III; the advanced stage refers to metastatic breast cancer, i.e., stage IV. Existing studies have shown that patients at different stages face significant differences in physical burden, treatment regimens, and expectations regarding life and death, all of which substantially influence their disease perception, emotional responses, and information processing strategies (Segrin et al., 2011).

Uncertainty Management Theory explains how individuals respond to situations filled with uncertainty (Brashers, 2001). It suggests that people do not always seek information when facing health-related threats. Instead, they selectively seek or avoid information often depends on how emotionally prepared they feel. This theory is especially relevant to cancer patients. As the disease progresses, their level of uncertainty often changes throughout the illness. These changes influence how they manage their emotions, also shape how patients choose to engage with health information. Moreover, the Kübler-Ross (1997). Model identifies five sequential psychological phases: denial, followed by anger, bargaining, later depression, finally acceptance. This progression typically occurs after life-altering diagnoses. We observe clear behavioral differences between early and late-stage patients. Those in early stages commonly display denial through two main behaviors: suppressing emotions and steering clear of alarming medical facts. In contrast, patients in advanced stages tend to demonstrate greater acceptance and are usually more willing to express their feelings openly.

Empirical evidence further supports the moderating role of cancer stage in patients' psychological and behavioral responses. Manne et al. (2001) showed that disease stage moderated the link between stress and avoiding health information, patients in early stages were more likely to avoid information. Chen and Zhao (2021) reported that patients with Stages I and II breast cancer tended to suppress their emotions, while stage IV patients showed more positive emotions and signaled a desire for connection with others. Segrin et al. (2011) also highlighted that patients at different stages show distinct emotional states, information processing patterns, and social engagement needs. Furthermore, Graham (2024) showed that disease stage moderated the association between psychological distress and health avoidance. The more advanced the disease, the more likely patients were to view such activities as emotional anchors and opportunities for meaning-making. According to IRCs theory (Collins, 2004), effective social interaction involves four key elements. These include bodily co-presence, a clear group boundary, a shared focus of attention, and emotional synchronization. Cancer patients at different stages respond to these elements in different ways. Based on the above facts, we predicted that the PES generated from ritual interactive activities would be more pronounced in enhancing positive emotions in advanced-stage patients. Therefore, the following hypothesis is proposed:

*H5. Cancer staging moderates the relationship between PES and positive emotions in patients with cancer*.

Cancer staging also affects perceived coping self-efficacy and subsequent health-promoting behaviors in patients with cancer (Hou et al., 2018; Taechaboonsermsak et al., 2005). These differences may influence how they process emotional experiences and engage in health-related behaviors, including HIA. Based on IRCs theory combination with Social Cognitive Theory, this study proposes Hypothesis H4, which explains how PES may reduce HIA through positive emotions and coping self-efficacy. However, the impact of this chain effect may differ depending on cancer staging. Patients in the early stages often pay more attention to clinical treatment and physical recovery. In contrast, those in advanced stages usually care more about making sense of their experience and seeking emotional support (Graham, 2024; Lee et al., 2008). Therefore, patients in more advanced stages may be more sensitive to emotional interactions and more likely to benefit from PES in ways that improve coping self-efficacy and reduce HIA. This analysis can be further characterized as a moderated chain mediation effect. Therefore, this study proposes the following hypothesis:

*H6. Cancer staging moderates the chain-mediated effects of patients' PES on HIA behavior via positive emotions and self-efficacy*.

**Figure 1:**
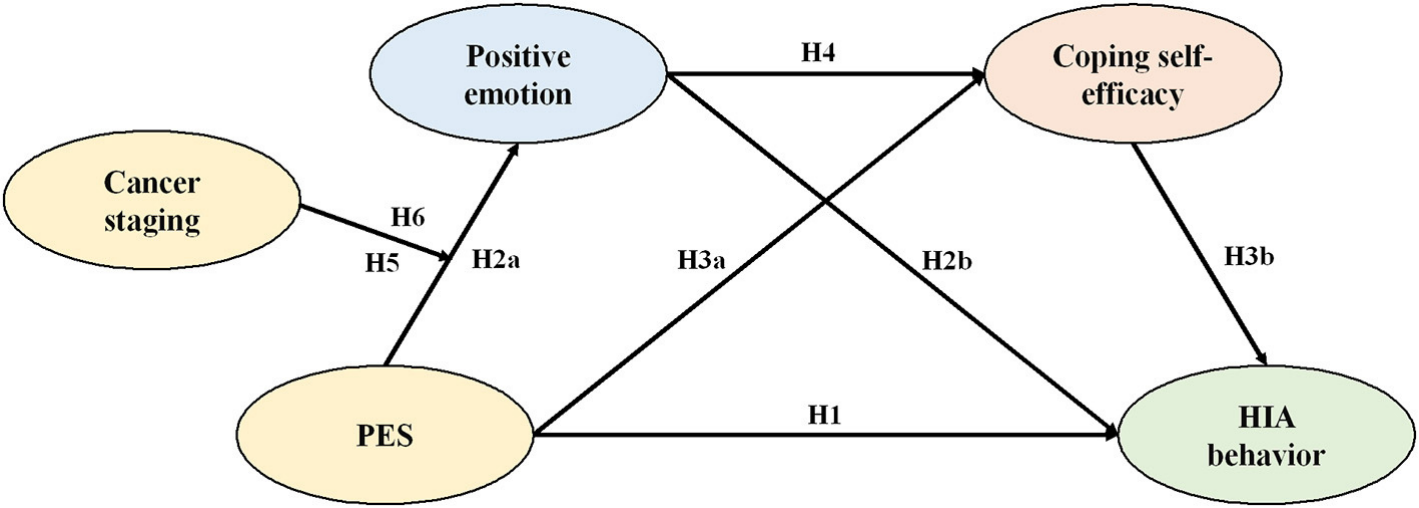
Model diagram of the present study.

This study constructed a moderated chain-mediated effects model (Figure 1) to comprehensively explore the mechanisms and boundaries of the role of PES on HIA behavior in patients with breast cancer.

## 2 Materials and methods

### 2.1 Participants and procedures

The study sample was drawn from five Grade A tertiary hospitals in China—three in Guizhou, one in Hebei, and one in Tianjin. The selected hospitals organized a series of patient interaction ritual activities irregularly, in addition to structured treatment. The main interactive activities include (i) ritual activities for traditional Chinese festivals, such as the Dragon Boat Festival; (ii) award ceremonies, such as the presentation of a “diploma” to a patient who has completed chemotherapy; (iii) outdoor rituals, such as patients' salon, anti-cancer star contests in “pink ribbon month”; and (iv) ceremonial interactions on special days, such as Women's Day and patients' birthdays. These interactions adhere to humanistic care and provide ritual and embodied experiences to the patients. These cases were therefore suitable for our study and reflected our research aims. The participants were all patients with breast cancer who were admitted to the above hospitals and who participated in interactive activities. After being informed of the study's goal and agreeing to take part in the study, the patients were asked to complete and submit the questionnaire. The survey data collected in this study has been strictly anonymized, and no identifying information is included.

**Figure 2:**
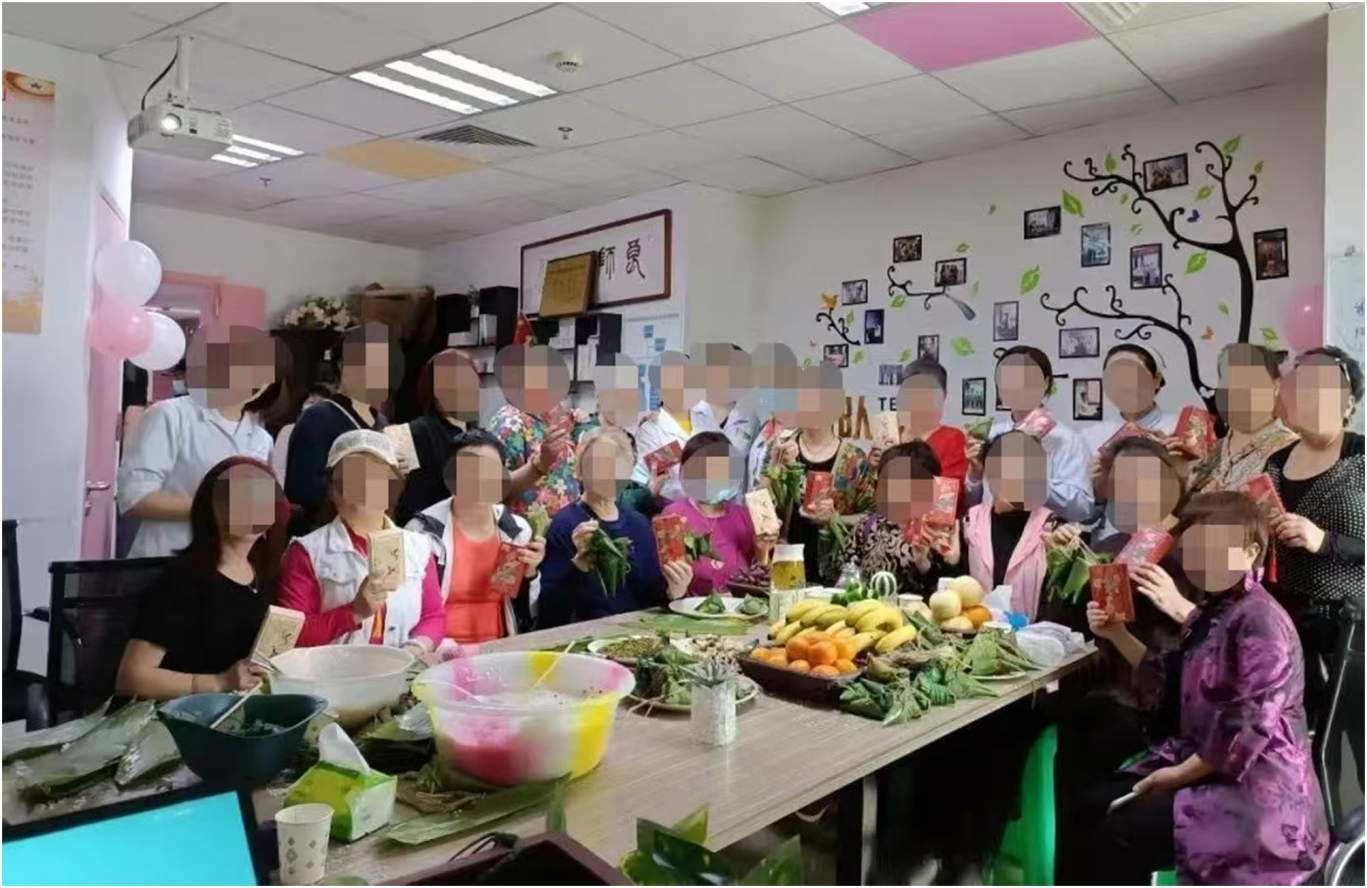
Patients participation in ritual activities for traditional Chinese festivals.

**Figure 3:**
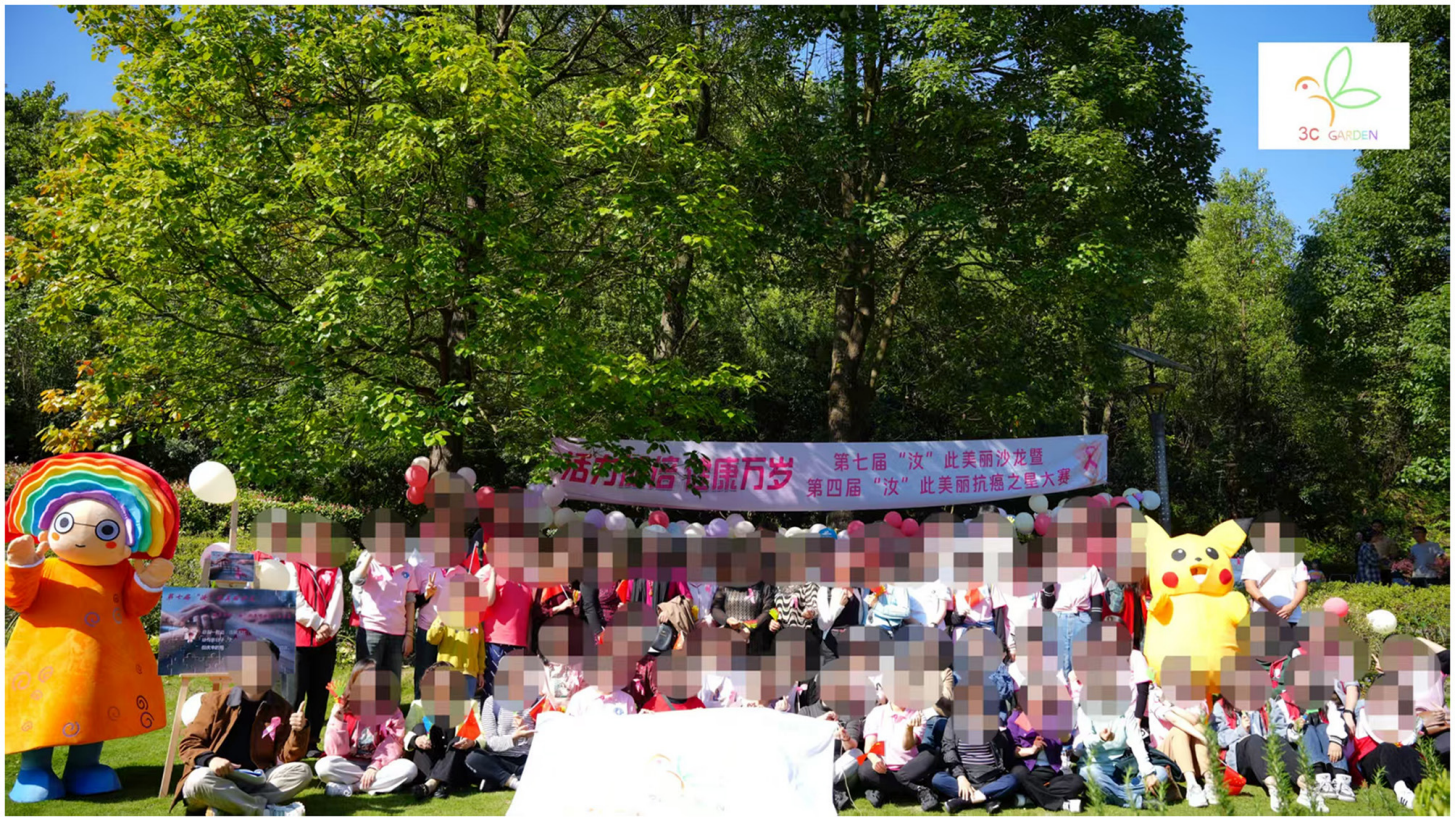
Patients participation in outdoor rituals interactive activities.

The survey was conducted over four periods. First, on June 21, 2023, research team members participated in the activities of the Breast Surgery Department of a tertiary hospital in Guizhou as volunteers, celebrating the Dragon Boat Festival with doctors and patients with breast cancer and conducting traditional rituals, such as wrapping zongzi and sewing scented sachets (Figure 2). In this survey, 18 questionnaires were distributed, and 18 were returned. Second, on October 15, 2023, research team members again voluntarily participated in an outdoor ceremony organized by a tertiary hospital in Guizhou—the Breast Cancer Patient Salon, and the Anti-Cancer Star Competition at a Healing Garden (Figure 3). In this survey, 43 questionnaires were distributed and 42 were returned. Third, on October 28, 2023, team members participated in an outdoor ceremony for Pink Ribbon Month, organized by a tertiary hospital in Guizhou. In this survey, 65 questionnaires were distributed and 60 were returned. Finally, we contacted three eligible hospitals in Tianjin, Hebei, and Guizhou following the Pink Ribbon Interactive Rituals that were held in the three hospitals, where 225 questionnaires were distributed and 217 were returned. In total, 337 questionnaires were retrieved. After excluding invalid questionnaires with too short a response time, consecutively choosing the same answer, and obvious patterns or errors in the answers, a total of 302 valid questionnaires were included in the analysis, an effective recovery rate of 89.61%. Owing to the specificity of breast cancer as a disease, all the valid samples were female patients with breast cancer, and the statistical information of the samples is shown in Table 1.

**Table 1:** Demographic statistics characteristics of samples.

**Category**	**Item**	**Percentage %**	**Category**	**Item**	**Percentage %**
Residence	Rural	22.8	Occupation	Civil servant	7.3
	Urban	76.8		Professionals & technicians	14.9
Age	21–30 years old	2.0		Personnel of enterprises	23.8
	31–40 years old	8.3		Business/service workers	6.6
	41–50 years old	35.4		Housewife	20.9
	51–60 years old	38.7		Others	26.5
	Over 61 years old	15.6	Cancer stage	Early	47.0
Education	High school and below	49.3		Mid-term	37.4
	Junior college	27.8		Advanced	15.6
	Bachelor degree	20.5	Surgical treatment	Yes	97.4
	Graduate degree	2.3		No	2.6
Monthly income	RMB 3,000 and below	46.0	Diagnosis duration	1 year or less	29.5
	RMB 3,001–6,000	33.4		2 years	24.5
	RMB 6,001–8,000	11.3		3 years	14.2
	RMB 8,001–10,000	3.0		4 years	10.6
	RMB 10,000 above	6.3		5 years and above	21.2

### 2.2 Measurements

All the measurement scales used in this study were in English, and have previously been published elsewhere. The PES scale was adopted from Páez et al., 2015. The Positive Emotion scale was derived from the scale (Diener et al., 2009). Coping self-efficacy was measured using the 12-item CBI-B (Heitzmann et al., 2011), which is a brief, validated scale with four factors and strong reliability (α = 0.84–0.88). The CBI-B is widely used in cancer research due to its efficiency and psychometric strength. The HIA scale was adapted from Howell and Shepperd (2017). The HIA scale used in this study was adapted from the original Information Avoidance Scale (IAS), developed by Howell and Shepperd (2016, 2017). Past studies have shown that reverse-worded items may confuse respondents or cause bias, especially in clinical groups or individuals under cognitive strain (Suárez-Álvarez et al., 2018; Weijters et al., 2013). To improve clarity and reliability, we excluded reverse-scored items and selected four IAS items to measure individuals' tendency to avoid health-related information. Patient demographic information was used as a control variable based on related research (Chen and Zhao, 2021; Ireland et al., 2019; Kübler-Ross, 1997).

First, we created a preliminary translation, invited experts in the field to review it, and discussed the content of translation, thus initially determining the Chinese measurement items. Two breast surgery experts were then invited to revise and adjust the questionnaire to include medically relevant questions. According to relevant studies (Perz et al., 2011; Segrin et al., 2011; Yao et al., 2015) and doctors' recommendations, cancer is classified into early, mid, and advanced stages. All items were measured on a five-point Likert scale, except for the control variables. A scale from “1” to “5” indicates “strongly disagree” to “strongly agree” respectively.

## 3 Data analysis and results

### 3.1 Homology bias test

In this study, we used Harman's one-way method, which employs principal component analysis without rotation to detect common method bias. The results of the exploratory factor analysis of the 26 question items showed that there were five factors with eigenvalues >1, cumulatively explaining 68.97% of the total variance; the first factor had a methodological explanatory rate of 16.90%, much <50%, indicating that the common methodological bias of this study was within the acceptable range and would not have a serious impact on the results of the study.

### 3.2 Reliability and validity test

As shown in Table 2, the Cronbach's alpha coefficients for the four variables in this study and the combined reliability CRs were greater than the commonly used criterion of 0.7. This indicates that the scales have good internal consistency and high reliability. Cluster validity can be tested based on standardized factor loadings and mean extracted variance of the variables (Fornell and Larcker, 1981). The standardized factor loadings for each of the question items in the four variables ranged from 0.56 to 0.88, all greater than the critical value of 0.5, and their average variances extracted (AVEs) were all greater than or equal to the recommended value of 0.5. This suggests that the variable scales have good convergent validity.

**Table 2:** Reliability and validity of the constructs.

**Constructs/items**		**Standard factor loading**	**α**	**CR**	**AVE**
**PES**			0.88	0.89	0.66
When participating in activities organized by hospitals or other institutions:					
PES1	It seemed to me as if we were a single person.	0.85			
PES2	We felt that we were one.	0.86			
PES3	We felt more sensitive to emotions and feelings others that feel.	0.71			
PES4	We felt a strong shared emotion.	0.82			
**Positive emotion**			0.92	0.92	0.66
When participating in activities organized by hospitals or other institutions:					
PE1	My emotion is good.	0.77			
PE2	My emotion is positive.	0.75			
PE3	My emotion is pleasant.	0.79			
PE4	My emotion is joy.	0.83			
PE5	My emotion is happy.	0.87			
PE6	My emotion is contented.	0.85			
**Coping self-efficacy**			0.92	0.92	0.50
SCE1	I can maintain independence.	0.60			
SCE2	I can maintain a positive attitude.	0.74			
SCE3	I can maintain a sense of humor.	0.67			
SCE4	I can express negative feelings about cancer.	0.56			
SCE5	I can maintain work activity.	0.62			
SCE6	I can remain relaxed throughout treatments and not allow scary thoughts to upset me.	0.68			
SCE7	I can actively participate in treatment decisions.	0.83			
SCE8	I can ask physicians questions.	0.86			
SCE9	I can seek consolation.	0.57			
SCE10	I can share feelings of concern.	0.59			
SCE11	I can manage nausea and vomiting.	0.81			
SCE12	I can cope with physical changes.	0.88			
**HIA**			0.88	0.88	0.65
HIA1	After cancer I would rather not know cancer.	0.79			
HIA2	I would avoid learning cancer during treatment.	0.73			
HIA3	When it comes to cancer, [sometimes] ignorance is bliss.	0.82			
HIA4	I can think of situations in which I would rather not know cancer.	0.87			

CR, composite reliability; AVE, average variance extracted.

Using Mplus 8.3, a validated factor analysis was conducted to evaluate the four main variables' discriminant validity. The four-factor model, displayed in Table 3, showed a good fit (χ2 = 663.68, df = 293, χ2/df = 2.27, <3; RMSEA = 0.065, < 0.08; SRMR = 0.042, < 0.05; TLI = 0.922, CFI = 0.930, both >0.9), indicating that the sample data of the present study and the four-factor model has a good matching effect, and the four-factor model outperforms alternative models, showing a better fit. Additionally, the square root of the mean extracted variance of each variable was higher than the other variables' correlation coefficient (Table 4). This demonstrates that the four key variables are different constructs with good discriminant validity in terms of connotations and measurements.

**Table 3:** Results of the confirmatory factor analyses.

**Models**	**χ^2^**	**df**	**χ^2^/df**	**SRMR**	**TLI**	**CFI**	**RMSEA**
Four-factor model (A, B, C, D)	663.68	293	2.27	0.042	0.922	0.930	0.065
Three-factor model (A, B + C, D)	1,375.71	296	4.65	0.081	0.775	0.795	0.110
Two-factor model (A + B + C, D)	1,729.67	298	5.80	0.086	0.704	0.728	0.126
One-factor model (A + B + C + D)	1,946.81	299	6.51	0.090	0.660	0.687	0.135

A indicates PES; B indicates positive emotion; C indicates coping self-efficacy; D indicates HIA; *N* = 302; IFI, incremental fit index; CFI, comparative fit index; RMSEA, root mean square error of approximation.

**Table 4:** Means, standard deviations, and correlations of all variables in this study.

**Variables**	**1**	**2**	**3**	**4**	**5**	**6**	**7**	**8**
1. Residence	–							
2. Age	0.23^**^	–						
3. Education	0.36^**^	0.07	–					
4. Cancer staging	−0.10^**^	−0.09	−0.19^**^	–				
5. PES	0.55	0.01	0.05	−0.05	**(0.81)**			
6. Positive emotion	0.13^*^	0.05	−0.01	−0.10	0.66^**^	**(0.81)**		
7. Coping self- efficacy	0.14^*^	0.10	0.06	−0.05	0.50^**^	0.59^**^	**(0.71)**	
8. HIA	−0.12^*^	−0.04	0.19^**^	0.14^*^	−0.56^**^	−0.63^**^	−0.62^**^	**(0.81)**
Mean	1.78	4.58	1.76	1.76	3.84	3.91	3.89	2.23
Standard deviation	0.44	0.92	0.86	0.89	0.59	0.62	0.57	0.69

*N* = 302.

^*^p < 0.05.

^**^p < 0.01.

In parentheses along the diagonal (in boldface) are the square roots of the average variance extracted.

### 3.3 Correlation analysis of the variables

Table 4 indicates a significant positive correlation between PES, positive emotions, and coping self-efficacy (*r* = 0.66, *p* < 0.01; *r* = 0.50, *p* < 0.01). Positive emotions were significantly and positively correlated with coping self-efficacy (*r* = 0.59, *p* < 0.01). Moreover, there was a significant negative correlation between PES, positive emotion, coping self-efficacy, and HIA behavior (*r* = −0.56, *p* < 0.01; *r* = −0.63, *p* < 0.01; *r* = −0.62, *p* < 0.01). The variables' correlation coefficients' signs and significance support the assumptions of the theoretical model and offer initial evidence for further hypothesis testing.

#### 3.3.1 Collinearity diagnostics by cancer stage

We checked for multicollinearity to ensure the stability of the moderated mediation model. We first ran linear regressions. PES and positive emotion were used to predict coping self-efficacy (CE). Both showed VIF values of 1. This suggested that there was no multicollinearity. Next, we dummy-coded the cancer stage variable. We then created interaction terms between PES and each dummy variable. These interaction terms were used to predict CE. All interactions were significant. None showed signs of serious multicollinearity. To further reduce potential bias, we mean-centered PES. We then regenerated the interaction terms. The results again showed no clear multicollinearity. This supported the model's robustness and interpretability.

### 3.4 Hypothesis testing

**Figure 4:**
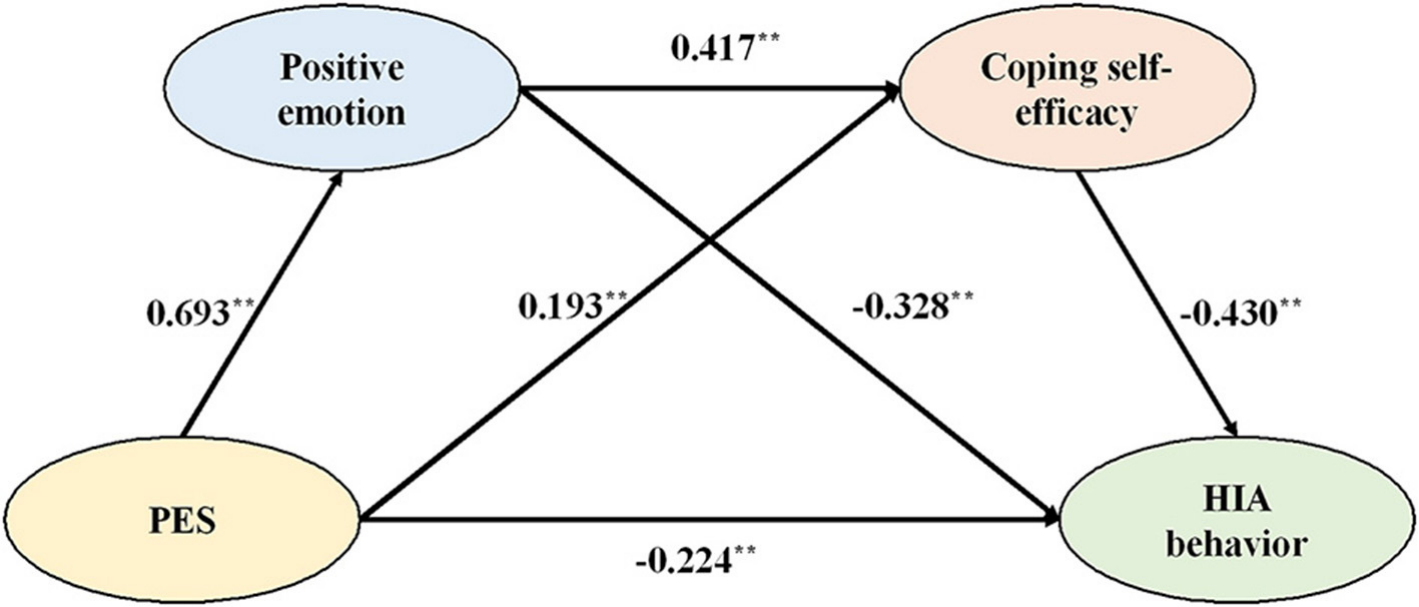
Path analysis diagram (*N* = 302). **indicates *p* < 0.01.

Hypothetical models were constructed using Mplus 8.3, to perform a path analysis and test the relationship between the variables. The best fit was found between the observed data and the hypothetical model, as indicated in Table 5. The results of path analysis using hypothetical model are shown in Figure 4.

**Table 5:** Model fitness index.

**Models**	**χ^2^**	**df**	**χ^2^/df**	**SRMR**	**TLI**	**CFI**	**RMSEA**
Model 1 (A-D)	43.435	19	2.286	0.025	0.975	0.983	0.065
Model 2 (A-B-D)	203.653	74	2.752	0.036	0.946	0.956	0.076
Model 3 (A-C-D)	442.319	167	2.649	0.044	0.917	0.927	0.074
Model 4 (A-B-C-D)	663.678	293	2.265	0.042	0.922	0.930	0.065

A indicates PES; B indicates positive emotion; C indicates coping self-efficacy; D indicates HIA.

With a standardized path coefficient of −0.224 (*p* < 0.01), PES was found to have a significant negative predictive effect on HIA. The standardized path coefficient of PES affecting positive emotions is 0.693 (*p* < 0.01), indicating that PES significantly and positively predicted positive emotions. Positive emotions, in turn, had a significant negative effect on HIA, as evidenced by the standardized path coefficient of −0.328 (*p* < 0.01) for positive emotions' impact on HIA. PES significantly positively predicted coping self-efficacy, as evidenced by the standardized path coefficient of PES affecting coping self-efficacy of 0.193 (*p* < 0.01). Coping self-efficacy had a significant negative predictive influence on HIA, with a standardized path coefficient of −0.430 (*p* < 0.01) for HIA. Moreover, positive emotions significantly positively predicted coping self-efficacy, as evidenced by the standardized path coefficient of positive emotions affecting coping self-efficacy of 0.417 (*p* < 0.01). Thus, H1, H2a, H2b, H3a, and H3b were approved.

Utilizing the PROCESS macro (Model 6), repeated bootstrap sampling was performed. The mediating role of positive emotions and coping self-efficacy on the relationship between PES and HIA was tested using 5,000 sampling repeats and a 95% confidence interval (CI). The outcomes are displayed in Table 6. Positive emotion had a significant mediating role between PES and HIA, the indirect impact value of positive emotion between PES on HIA was −0.227, and the 95% CI (−0.344, −0.101) did not contain 0; therefore, H2 is supported. Coping self-efficacy had a significant mediation effect between PES and HIA, as evidenced by the indirect effect value of −0.083 of coping self-efficacy between PES on HIA and the absence of 0 in the 95% CI (−0.157, −0.023). H3 is therefore supported. Positive emotions and coping self-efficacy had a significant chain-mediating effect, the value of this effect was −0.124, and the 95% CI (−0.202, −0.060) did not contain 0. H4 is therefore supported.

**Table 6:** Bootstrap test of mediating effects with R^2^ and F-statistics.

**Effect**	**Path**	**Effect value**	**Boot SE**	**Bootstrap 95% CI**	***R* ^2^**	**F (df1, df2)**	***p***
Indirect effect	Ind1	−0.227	0.062	(−0.344, −0.101)	0.469	F (10, 291) = 25.683	*p* < 0.01
	Ind2	−0.083	0.035	(−0.157, −0.023)	0.502	F (10, 291) = 29.286	*p* < 0.01
	Ind3	−0.124	0.037	(−0.202, −0.060)	0.545	F (11, 290) = 31.509	*p* < 0.01
Total indirect effect		−0.434	0.059	(−0.547, −0.314)	

ind1: PES → Positive emotion → HIA; ind2: PES → Coping self-efficacy → HIA; ind3: PES → Positive emotion → Coping self-efficacy → HIA.

In addition to the indirect effect values, Table 6 also reports the model fit statistics (R^2^ and F values) for each mediation path. The explained variance (R^2^) of the three models ranges from 0.469 to 0.545, and all F-tests reached a significant level (*p* < 0.01), indicating that the models fit well and have strong explanatory power.

To ensure that cancer staging was a suitable moderator, we first tested for group homogeneity. One-way ANOVA and post hoc LSD tests were conducted for PES and CE (Coping Self-Efficacy). The results showed no significant group differences for PES (F = 0.418, *p* = 0.740) or CE (F = 0.706, *p* = 0.549). Pairwise comparisons also showed no significant differences between any two groups (all *p* > 0.05). These findings indicate that the variables were evenly distributed across cancer stages. This satisfies the statistical assumption required for moderation analysis. As Hayes (2018) points out, a moderator does not need to have a significant main effect. Its value lies in how it changes the strength or direction of the predictor–outcome relationship. Based on this, we proceeded to test cancer staging as a moderator in the model.

**Figure 5:**
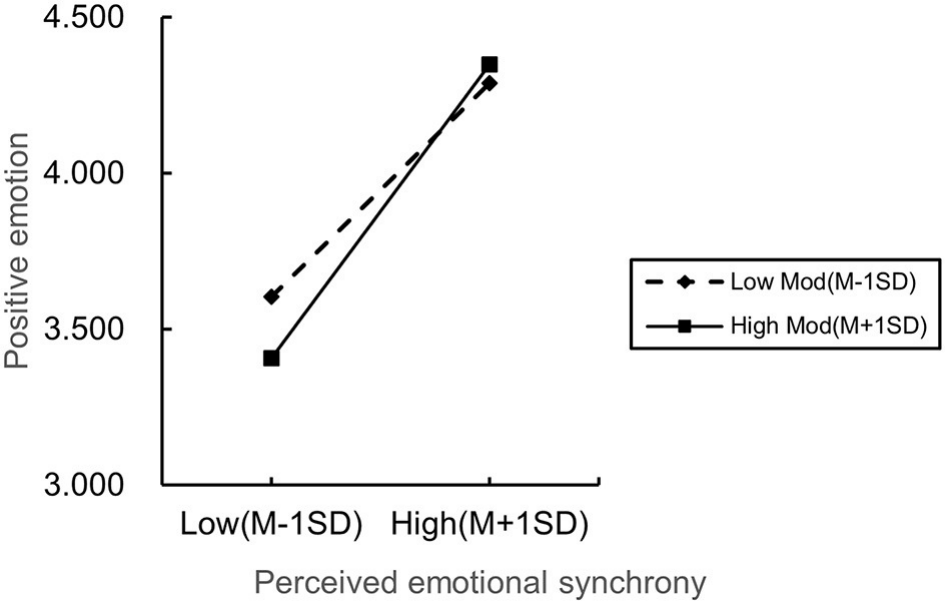
Cancer staging adjustment effect.

PROCESS macro (Model 83) was used to test the moderating impact of cancer staging. The results were as follows: First, positive emotion was significantly impacted by the interaction term between PES and cancer staging (β = 0.131, *p* < 0.05), indicating that the relationship between PES and positive emotion was significantly moderated by cancer staging. A moderating effect analysis was conducted to further explain the relationship between the moderating effects (Figure 5). The results showed that when the stage of disease progression was in a high subgroup (tending to advanced breast cancer), the positive effect of PES on positive emotion was stronger [β = 0.791, 95% CI = (0.674, 0.9090), *p* < 0.05]; for patients in the low subgroup of disease progression stage (tending toward early breast cancer), the positive effect of PES on positive emotion was weaker, but still reached the significance level [β=0.576, 95% CI = (0.452, 0.699), *p* < 0.05]. This indicates that the positive effect of PES on positive emotions is enhanced when the stage of disease progression tends to advance, supporting H5.

Second, in the chain mediation of patients' PES through positive emotions and coping self-efficacy with HIA, the product of the path coefficients between the interaction and mediating variables was −0.024 (*p* < 0.05), suggesting that the chain mediation effect was moderated by cancer staging. In Table 7, when the cancer staging was in the low subgroup (trending toward early stage), the mediation effect value of patients' PES through positive emotions and coping self-efficacy to HIA was [β = −0.189, 95% CI = (−0.294, −0.082), *p* < 0.05], with a significant chain-mediated effect; When the cancer staging was in a high subgroup (trending toward advanced stage), the mediation effect value of patients' PES through positive emotions and coping self-efficacy to HIA was [β = −0.259, 95% CI = (−0.388, −0.1160), *p* < 0.05], with a significant chained mediation effect; at both the high and low levels, the indirect effect differed significantly, with a difference of −0.071 [95% CI = (−0.147, −0.013), *p* < 0.05]. This indicates that the chain-mediated effects of positive emotions and coping self-efficacy between PES and HIA are significantly strengthened among patients with advanced-stage breast cancer. In addition to the full chain mediation, we also examined two simpler mediation paths (Table 8). These were: PES → positive emotion → HIA, and PES → coping self-efficacy → HIA. In Table 8, we tested whether cancer staging moderated these two indirect effects. For the first path (PES → PE → HIA), the indirect effect was significant in both early-stage and advanced-stage groups. However, it was stronger in the advanced-stage group [β = −0.401, 95% CI = (−0.516, −0.276)] than in the early-stage group [β = −0.292, 95% CI = (−0.390, −0.191)]. The index of moderated mediation was −0.067 [95% CI = (−0.131, −0.012)], which confirms that cancer staging significantly influenced this mediation pathway. For the second path (PES → CE → HIA), a similar trend was found. The indirect effect was −0.335 [95% CI = (−0.462, −0.204)] in the advanced-stage group, and β = −0.189 [95% CI = (−0.302, −0.094)] in the early-stage group. The index of moderated mediation was −0.089 [95% CI: (−0.157, −0.011)], which again indicates a significant moderation effect. These results demonstrate that cancer staging affects the full chain of mediation and shapes the strength of each individual mediation path. In both cases, patients at more advanced stages experienced stronger indirect effects. In summary, being in different disease stages significantly moderated the magnitude of the chain-mediated effect, supporting H6.

**Table 7:** Analysis of moderated chain mediation effects.

**Moderator variable**	**Path: PES**→**Positive emotion**→**Coping self-efficacy**→**HIA behavior**	
	**Effect value**	**95% CI**
Tendency to early staging	−0.189	(−0.294, −0.082)
Tendency to advanced staging	−0.259	(−0.388, −0.116)
Disparity	−0.071	(−0.147, −0.013)

**Table 8:** Results of the moderated-mediation effect.

**Cancer staging value**	**PES (X)**→**Positive emotion (M1)**→**HIA (Y)**								**PES (X)**→**Coping self-efficacy (M2)**→**HIA (Y)**							
	**Indirect effect**	**Boot SE**	**95%Boot**		**Index**	**Boot SE**	**95%Boot**		**Indirect effect**	**Boot SE**	**95%Boot**		**Index**	**Boot SE**	**95%Boot**	
			**Boot LLCI**	**Boot ULCI**			**Boot LLCI**	**Boot ULCI**			**Boot LLCI**	**Boot ULCI**			**Boot LLCI**	**Boot ULCI**
Low cancer staging (−1 S.D.)	−0.292	0.051	−0.390	−0.191	−0.067	0.030	−0.131	−0.012	−0.189	0.053	−0.302	−0.094	−0.089	0.037	−0.157	−0.011
M (0.00)	−0.342	0.050	−0.437	−0.238					−0.257	0.050	−0.360	−0.162				
High cancer staging (+1 S.D.)	−0.401	0.061	−0.516	−0.276					−0.335	0.065	−0.462	−0.204				

## 4 Discussion

### 4.1 Theoretical implications

The theoretical implications of this study are as follows. First, this quantitative study proposes a conceptual model to explain the reduction of HIA from the perspective of PES in cancer patients during ritual interactions, utilizing the theoretical frameworks of IRCs and social cognition. Existing studies have argued that interpersonal interactions between patients are of significant value (Birkelund and Larsen, 2013), and that peer patients are the best information providers, because of their similar physical experiences (Kulik et al., 2000). Previous studies have rarely explored the mechanisms influencing HIA by considering the PES factors in patient interactions. This study argues that applying the IRCs theory to the enhancement of positive emotions and coping self-efficacy by emotional energy in patients' ritual interactions is also useful in explaining how and why patients with cancer reduce HIA.

Second, we explored a chain mediation mechanism linking PES to HIA through two mediators: positive emotions and coping self-efficacy. The results suggest that PES exerts an indirect effect on HIA through these two factors, in which positive emotions and coping self-efficacy act as separate or linked mediators. Previous research has typically focused on positive emotions and coping self-efficacy as the most direct antecedents (Chasiotis et al., 2021; Hua and Howell, 2022), ignoring the mechanisms by which the two are transmitted between PES and HIA. In contrast, this study integrates them into a unified chain mediation model, offering a more comprehensive understanding of their sequential roles. We conducted internal model comparisons among three competing models: a chain mediation model including both mediators in sequence, and two alternative models each including only one mediator. The chain model demonstrated superior model fit (e.g., CFI = 0.930, RMSEA = 0.065; see Table 5) and a higher R^2^ for HIA (R^2^ = 0.545; see Table 6), indicating stronger explanatory power in predicting patients' HIA behavior. Drawing on this chain structure, we identified four possible pathways linking PES and HIA. These findings expand our understanding of how PES works during ritual interactions. They also offer a stronger theoretical foundation for future research on patients' shared emotional experiences.

Third, this study emphasizes the importance of cancer staging as an individual difference that influences emotional and behavioral responses. Considering that disease progression affects patient emotions (Chen and Zhao, 2021; Kübler-Ross, 1997), this study verified the moderating effect of cancer staging on the mediator model, illustrating the interaction between cancer staging and PES on positive emotions and HIA behavior to varying degrees, thereby enriching the theoretical results. By integrating cancer staging into the model, this study extends current theories of health information avoidance. This dynamic perspective deepens our understanding of PES and offers a more nuanced framework for examining patient behavior in cancer care contexts.

### 4.2 Practical implications

First, PES in peer interactions among breast cancer patients should be actively promoted. Consistent with what Zlobina and Dávila (2022) emphasized, collective PES experiences can lead to positive health behaviors. This study confirms that PES generated in ritual interactions significantly influences patients' HIA behaviors. Given that the majority of breast cancer patients are women—who are generally more receptive to emotionally focused supportive communication (Kunkel and Burleson, 1999), empathy-centered support programs are especially valuable. Hospitals, community organizations, and NGOs should co-develop structured ritual-based group activities (e.g., healing circles, commemorative events, shared creative activities like planting or painting) to promote emotional sharing and trust. Furthermore, clinical staff and patient navigators can play an active role in inviting new patients to these groups through digital platforms, posters in clinics, and direct referrals from physicians.

Second, ritual interaction activities are an effective method of clinical intervention. Consistent with previous studies, ritual activities play a positive role in cancer treatment (Santos et al., 2018; Buchbinder et al., 2009). Therefore, the following ritual interactive activities could be considered for integration into clinical intervention practices: celebrations of traditional Chinese festivals; award ceremonies, such as presenting a “diploma” to patients who have completed chemotherapy; outdoor rituals like patient salons and “anti-cancer star” contests during Pink Ribbon Month; and ceremonial interactions on special occasions such as Women's Day and patients' birthdays.

Third, the cancer stage should be considered when implementing health interventions. This study found that the cancer staging was a particularly important moderating variable, consistent with the findings of Chen and Zhao (2021), patients in Stages I and II were more likely to suppress their emotions. Therefore, it is important for clinicians to implement personalized interventions based on the cancer stage to better promote health information behaviors. For early-stage patients, small-scale expressive interaction activities guided by clinical nurses are organized to help build a sense of safety and alleviate information-related anxiety. For mid-stage patients, patient support meetings are held to stimulate their initiative and enhance self-efficacy. For late-stage patients, carefully designed commemorative rituals affirm their perseverance in treatment and promote the construction of life meaning.

### 4.3 Limitations and future studies

This study has the following limitations. First, we made no distinctions between geographical differences. The research found that there are differences in the form and effect of patient interaction activities and emotional energy in large hospitals in China's eastern, central, and western cities. In the future, it will be possible to differentiate them according to geographic regions and to compare the differences in the formation mechanism of patients' emotional energy on HIA behavior. Second, this study did not consider the status of the participants. According to IRCs theory, under the status rituals, leaders in the central position or high status of participants have the highest emotional energy, while followers in the periphery of the status of the follower obtain less emotional energy (Collins, 2004). Third, this study explored the mechanism of influence between PES and HIA in patients' ritual interactions, mainly at the individual level, without considering the different forms of ritual interaction. In reality, this should be a complex process with multiple levels and factors. In the future, we can continue to explore other mediating variables that have not been the focus of this study, from the organizational and geographical levels, to continuously improve the theoretical findings from multiple perspectives. Finally, although this study focuses on the maladaptive aspects of HIA, it is important to acknowledge that HIA may also serve as an adaptive coping strategy under specific emotional or contextual conditions. Future studies should pay attention to the situational and adaptive dimensions of HIA, especially regarding how patients balance information engagement with psychological self-protection.

## 5 Conclusions

Using IRCs and social cognitive theories as a framework, this study proposes a first-stage moderated chain mediation model to investigate the effects of PES on patients' ritual interaction with HIA, with positive emotions and coping self-efficacy as the mediators and cancer staging as the moderator. Our research suggests that PES in the ritual interactions of patients with breast cancer was negatively related to HIA via positive emotions and coping self-efficacy. Our findings highlight the importance of adopting an interactional ritual perspective in understanding patient interactions. Guided by the IRCs theory, the physical presence of other patients with cancer while participating in ritualistic interactive activities allows them to feel emotional energy and positive attitudes, which contribute to their health information behaviors.

Furthermore, our findings show that cancer staging is a regulatory element that moderates HIA reactions to patient PES. Clinicians should implement personalized interventions based on cancer staging to better promote health information behaviors and provide more humane care for patients with breast cancer to stimulate emotional energy, thereby reducing the occurrence of HIA.

## Data Availability

The raw data supporting the conclusions of this article will be made available by the authors, without undue reservation.
